# Moving Forward With Telehealth in Cancer Rehabilitation: Patient Perspectives From a Mixed Methods Study

**DOI:** 10.2196/46077

**Published:** 2023-11-09

**Authors:** Linda O'Neill, Louise Brennan, Grainne Sheill, Deirdre Connolly, Emer Guinan, Juliette Hussey

**Affiliations:** 1 Discipline of Physiotherapy School of Medicine Trinity College Dublin, the University of Dublin Dublin 8 Ireland; 2 Trinity St James's Cancer Institute Dublin Ireland; 3 Discipline of Occupational Therapy School of Medicine Trinity College Dublin, the University of Dublin Dublin Ireland

**Keywords:** telehealth, telemedicine, cancer rehabilitation, oncology, qualitative, mixed methods, mobile phone

## Abstract

**Background:**

The COVID-19 pandemic accelerated the use of telehealth in cancer care and highlighted the potential of telehealth as a means of delivering the much-needed rehabilitation services for patients living with the side effects of cancer and its treatments.

**Objective:**

This mixed methods study aims to explore patients’ experiences of telehealth and their preferences regarding the use of telehealth for cancer rehabilitation to inform service development.

**Methods:**

The study was completed in 2 phases from October 2020 to November 2021. In phase 1, an anonymous survey (web- and paper-based) exploring the need, benefits, barriers, facilitators, and preferences for telehealth cancer rehabilitation was distributed to survivors of cancer in Ireland. In phase 2, survivors of cancer were invited to participate in semistructured interviews exploring their experiences of telehealth and its role in cancer rehabilitation. Interviews were conducted via telephone or video call following an interview guide informed by the results of the survey and transcribed verbatim, and reflexive thematic analysis was performed using a qualitative descriptive approach.

**Results:**

A total of 48 valid responses were received. The respondents were at a median of 26 (range 3-256) months after diagnosis, and 23 (48%) of the 48 participants had completed treatment. Of the 48 respondents, 31 (65%) reported using telehealth since the start of the pandemic, 15 (31%) reported having experience with web-based cancer rehabilitation, and 43 (90%) reported a willingness for web-based cancer rehabilitation. A total of 26 (54%) of the 48 respondents reported that their views on telehealth had changed positively since the start of the pandemic. Semistructured interviews were held with 18 survivors of cancer. The mean age of the participants was 58.9 (SD 8.24) years, 56% (10/18) of the participants were female, and 44% (8/18) of the participants were male. Reflexive thematic analysis identified 5 key themes: telehealth improves accessibility to cancer rehabilitation for some but is a barrier for others, lived experiences of the benefits of telehealth in survivorship, the value of in-person health care, telehealth in cancer care and COVID-19 (from novelty to normality), and the future of telehealth in cancer rehabilitation.

**Conclusions:**

Telehealth is broadly welcomed as a mode of cancer rehabilitation for patients living with and beyond cancer in Ireland. However, issues regarding accessibility and the importance of in-person care must be acknowledged. Factors of convenience, time savings, and cost savings indicate that telehealth interventions are a desirable patient-centered method of delivering care when performed in suitable clinical contexts and with appropriate populations.

## Introduction

### Background

Telehealth has been widely adopted as an effective way to provide health care and continue access to a vast range of clinical specialties since the beginning of the COVID-19 pandemic [1-4]. Before this, telehealth, that is, the provision of health care at a distance using information and communication technology [5], was not widely used, despite being in existence for several decades [4,6]. Although the sudden and widespread adoption of telehealth in 2020 enabled the continued provision of health care, it also fueled an investment in digital infrastructure, regulatory changes, and innovations in care, creating an ideal environment for its continued growth [7-9]. Emerging literature suggests that there is a role for telehealth beyond the pandemic to enhance patient outcomes and improve convenience, efficiency, and access to care [8,10,11].

Cancer rehabilitation aims to reduce the physical, psychosocial, and cognitive effects of cancer and its treatment on patients through specialist input from health care professionals, including physiotherapists, psycho-oncologists, exercise physiologists, dietitians, and occupational therapists [12]. Many cancer rehabilitation services that were previously delivered in person swiftly pivoted to telehealth models of delivery at the beginning of the pandemic. Telehealth was found to be acceptable and feasible in cancer rehabilitation [1], and it offers several advantages to patients, including reduced travel time, improved access to those where geographical distance previously precluded participation, reduced costs, and greater convenience, indicating that telehealth can be a valuable, patient-centered mode of service delivery once it is appropriately implemented [13,14].

However, there are challenges associated with telehealth in cancer rehabilitation. Some patients require, or have a strong preference for, in-person care; equally, certain rehabilitation interventions can be unsuitable for, or compromised through, web-based delivery [13,15,16]. In addition, there are important issues regarding equality and inclusion to address. Although telehealth facilitates access in some cases, there are many groups for whom telehealth would impair access, such as those with poor internet connectivity or lower IT skills [17-19]. Many factors influence telehealth access and use, and throughout the pandemic, telehealth was found to be better adopted by those of a younger age, those with higher levels of education, and those living in urban areas [20,21].

As we emerge from the COVID-19 pandemic and health care services return to in-person models of delivery, we have a new awareness of the capability of telehealth to transform health care. Using this new information, we can harness the benefits of telehealth to develop and improve cancer rehabilitation services on national and international levels. The focus of service improvements should always be on providing high-quality care, which is accessible and safe, and be built around the needs and preferences of patients [6,22,23]. This can be achieved by first understanding patient experiences and preferences of telehealth in cancer rehabilitation and then applying this knowledge to co-design suitable services [24]. In early 2020, when many cancer rehabilitation services urgently changed from in-person to telehealth, there was no time to discuss with stakeholders how best to make this change. We now have the opportunity to consult with people living with and beyond cancer and gather recommendations for telehealth in cancer rehabilitation; this process has been commenced across other rehabilitation specialties, including cardiac and stroke rehabilitation [20,25].

### Objectives

The aim of this study is to understand patient experiences of and preferences for telehealth for cancer rehabilitation, with a view to making recommendations for the development of cancer rehabilitation services in a postpandemic health care system.

## Methods

### Overview

A mixed methods approach was implemented across 2 methodological phases to enable an in-depth exploration of the patients’ experiences of and preferences for telehealth delivery of cancer rehabilitation. In phase 1, a national survey was conducted to investigate the need, benefits, barriers, facilitators, and preferences for telehealth cancer rehabilitation. In phase 2, using a qualitative methodology, semistructured interviews were conducted to explore patients’ experiences of and preferences for cancer rehabilitation via telehealth in greater depth.

### Phase 1: Survey

#### Study Design and Participants

In phase 1, people living with and beyond cancer from across Ireland were invited to complete an anonymous survey (eg, web- and paper-based). The exclusion criterion was no history of cancer diagnosis.

#### Survey Instrument

The survey instrument was developed by a team of 4 researchers (LON, GS, EG, and DC) with expertise in cancer rehabilitation in partnership with 3 patient representatives who advised on the content and usability and piloted and approved the finalized survey. The final survey consisted of 25 questions, including 24 closed questions (including dropdown questions, a rating scale, and dichotomous questions [yes or no options]) and 1 open-ended question (ie, qualitative data), which were split across 3 sections. Section 1 gathered demographic information including age group, gender cancer diagnosis, and treatment. Section 2 asked participants to identify the side effects associated with their cancer and its treatments and their needs for rehabilitation. Section 3 explored (1) previous use of telehealth; (2) willingness to use telehealth for cancer rehabilitation; (3) barriers, benefits, and facilitators of telehealth; (4) preferences for the format of cancer rehabilitation via telehealth; and (5) how the COVID-19 pandemic has influenced their views on telehealth.

#### Data Collection and Analysis

Data were collected over a 2-month period between October and November 2020 using a voluntary sampling process. The survey was administered on the web through the XM survey software tool (Qualtrics) and circulated through the social media platforms of the Trinity St James’s Cancer Institute (TSJCI) and associated clinical and academic partners, by charity partners (eg, the Oesophageal Cancer Fund and Irish Cancer Society), and through our national cancer agency the National Cancer Control Program. Paper versions of the survey were provided to patients attending physiotherapy outpatient appointments at the TSJCI, Ireland’s largest cancer center.

Categorical data analysis was performed using Microsoft Excel, and the results were presented as counts and percentage frequency of responses. The responses to the open-ended question regarding the impact of COVID-19 on telehealth views were evaluated using content analysis by 2 researchers (LON and LB), who coded the responses and then grouped the responses into key findings.

### Phase 2: Semistructured Interviews

#### Study Design and Participants

Phase 2 used qualitative methodology (ie, semistructured interviews) to gain deeper insights and understanding of the role of telehealth in the delivery of cancer rehabilitation. The inclusion criteria stated that adults with a confirmed diagnosis of cancer living in Ireland were eligible to participate. A voluntary sampling method was applied, in which participants in phase 1 were invited upon completion of the survey to express an interest in participation in phase 2. In addition, an advertisement seeking participants was circulated through the social media platforms of the TSJCI and associated clinical and academic partners, charity partners, and the National Cancer Control Program. Recruitment persisted until researchers determined that the data had reached a level of depth where no new themes or codes were emerging and the study could be reproduced [26,27]. The interviews were conducted and reported according to the Consolidated Criteria for Reporting Qualitative Research checklist for qualitative studies [28].

#### Data Collection and Analysis

Sociodemographic information and details pertaining to the current use of technology were reported by the participants. A total of 3 female specialist cancer rehabilitation physiotherapists (LON, LB, and GS [all recipients of PhD in the field of cancer rehabilitation]) who were experienced in qualitative research with patients living with and beyond cancer conducted the one-on-one interviews. Most participants (16/18, 89%) had no previous engagement with the research team, and 11% (2/18) of the participants had participated in previous research projects at this center. Semistructured interviews followed a flexible interview guide (Textbox 1), which was developed by a team of 4 researchers (LON, GS, EG, and DC) in partnership with our patient representatives. After completion of the survey, the interview guide was refined to address the findings and topics of interest from the survey. The interview guide explored the participants’ previous experiences of telehealth and their perspectives on its role in cancer rehabilitation. Interviews were conducted remotely via telephone or video call, were audio recorded, and transcribed verbatim. The participants were not given the transcripts for their input or feedback.

**Phase 2 semistructured interview guide.**

**Phase 2 semistructured interview guide questions**

1. What is your overall impression of telehealth?
2. What do you think are the advantages and disadvantages of delivering health care in this way?
3. Can you describe your experience of receiving health care through telehealth?
4. What role can telehealth play in providing cancer rehabilitation services?
5. Do you have any suggestions for how telehealth could be used to help support people during and after cancer treatment?
6. Do you think the COVID-19 pandemic has changed patients’ view of telehealth? Can you describe how?

Phase 2 transcripts were imported into the NVivo (Lumivero) qualitative data analysis management software. Reflexive thematic analysis was performed using a qualitative descriptive approach [29] by 2 researchers (LON and LB) following the standardized process described by Braun and Clarke [30,31]. After a period of data familiarization, codes were generated across the data set and grouped into themes. The 2 researchers compared their codes and themes generated, and any differences in coding were resolved through consensus to determine the final themes and codes.

### Ethical Considerations

Ethics approval for human participant research was granted by the Tallaght University Hospital and St James’s Hospital Research Ethics Committee, Dublin, Ireland (REC:2020-07 List 25-Amendment 23). The study was conducted in accordance with the Declaration of Helsinki, and all participants provided informed consent (written or via electronic form) before undertaking the survey and the semistructured interview. To protect the privacy and confidentiality of the participants, phase 1 data were anonymous, and phase 2 data were pseudonymized. Participants received no compensation monetary or otherwise for their participation.

## Results

### Phase 1: Survey

A total of 48 valid responses to the survey were obtained, 44 (92%) of which were submitted on the web. Demographics and cancer-related characteristics are presented in Table 1.

**Table 1:** Survey participant demographics and cancer-related characteristics (N=48).

Characteristic		Values
**Age (years), n (%)**		
	18-24	1 (2)
	25-34	3 (6)
	35-44	11 (23)
	45-54	24 (50)
	55-64	4 (8)
	65-74	5 (10)
	>75	0 (0)
**Gender, n (%)**		
	Female	36 (75)
	Male	11 (23)
	Nonbinary	1 (2)
Time since cancer diagnosis (months), median (range)		26 (3-256)
**Cancer type, n (%)**		
	Breast	26 (54)
	Esophageal	6 (13)
	Bladder	2 (4)
	Lung	2 (4)
	Ovarian	2 (4)
	Prostate	2 (4)
	Other	8 (17)
Diagnosis of metastatic cancer, n (%)		11 (23)
**Cancer treatment received, n (%)**		
	Surgery	41 (85)
	Chemotherapy	32 (67)
	Radiation therapy	33 (69)
	Immunotherapy	5 (10)
	Stem cell therapy	1 (2)
	Hormone therapy	18 (38)
	Targeted therapy	3 (6)
	Alternative therapy	1 (2)
**Treatment status, n (%)**		
	Treatment completed	23 (48)
	Treatment ongoing	25 (52)
**Cancer and treatment side effects, n (%)**		
	Participants reporting side effects	41 (85)
	Participants reporting ≥3 side effects	34 (71)
	Participants who would like help with side effects	37 (77)

The participants were mostly female (36/48, 75%) and aged <55 years (39/48, 81%), and breast cancer was the most common diagnosis (26/48, 54%). Most of the participants (41/48, 85%) reported experiencing ongoing side effects of their cancer and treatment, and 71% (34/48) of the participants reported experiencing ≥3 side effects. The most frequent side effects were fatigue (33/48, 69%), pain (24/48, 50%), menopausal issues (19/48, 40%), anxiety (18/48, 38%), and nerve problems such as numbness and tingling (18/48, 38%). In total, 77% (37/48) of the participants reported feeling that they could benefit from seeing a health care professional regarding their side effects.

The respondents’ perceptions of telehealth including ease of use, benefits, and barriers are presented in Table 2.

In total, 31 (65%) of the 48 respondents had experienced telehealth since the onset of the COVID-19 pandemic, and most of the respondents (43/48, 90%) were open to using it specifically for cancer rehabilitation. Furthermore, 26 (54%) of the 48 respondents reported that the COVID-19 pandemic had changed their views on telehealth. Content analysis of open-ended responses revealed that the pandemic required people to become more familiar with videocalls (in multiple aspects of life). Participants felt that telehealth was a safe way to access health care services during this time. Some participants were now more likely to engage in telehealth, even those who had not used it before. A small proportion of respondents (5/48, 10%) reported frustrations because of the lack of in-person contact during the pandemic. Respondents outlined preferences for future delivery of cancer rehabilitation via telehealth, and these findings have been synthesized with preferences noted in the phase 2 semistructured interviews.

**Table 2:** Survey participants’ experiences and perceptions of telehealth (N=48).

Telehealth-related question			Values
**Used telehealth for medical or rehabilitation purposes during the COVID-19 pandemic, n (%)**			
	Yes	31 (65)	
	No	17 (35)	
**Type of medical, rehabilitation, or support service accessed via telehealth, n (%)**			
	Hospital consultant	22 (46)	
	GP^a^ appointment	16 (33)	
	1:1 health care professional appointment	8 (17)	
	Exercise class	16 (33)	
	Mindfulness session	11 (23)	
	Relaxation session	4 (8)	
	Other	8 (17)	
**Reported ease of access to telehealth, median (range)**			
	Ease of use rated on a scale ranging from 0=difficult to 10=very easy	8 (0-10)	
**Accessed telehealth cancer rehabilitation services, n (%)**			
	Yes	15 (31)	
	No	33 (68)	
**Willing to access telehealth cancer rehabilitation services, n (%)**			
	Yes	43 (90)	
	No	5 (10)	
**Has COVID-19 changed your views on telehealth?** **n (%)**			
	Yes	26 (54)	
	No	22 (46)	
**Perceived benefits of telehealth, n (%)**			
	Time saved	38 (79)	
	Cost saved	31 (65)	
	Reduced waiting time	33 (69)	
	Reduced face-to-face interaction	25 (50)	
	Other	4 (8)	
**Perceived barriers to patients’ use of telehealth, n (%)**			
	Difficulty with internet access	7 (15)	
	Poor IT skills	2 (4)	
	Web-based security concerns	5 (10)	
	Do not like using digital technology for health	5 (10)	
	Other	6 (13)	
**Perceived facilitators to patients’ use of telehealth, n (%)**			
	Device provision	6 (13)	
	Internet provision	4 (8)	
	Introductory telehealth call	15 (31)	
	Introductory in-person session	27 (56)	
	Telehealth hotline	18 (38)	
	Other	4 (8)	

^a^GP: general practitioner.

### Phase 2: Semistructured Interviews

#### Overview

A total of 18 people with a history of cancer participated in phase 2 interviews. The median interview duration was 21 (range 7-46) minutes. Participant sociodemographic data are presented in Table 3.

**Table 3:** Semistructured interviews—sociodemographic characteristics (N=18).

Participant number	Gender	Age (years), range	Cancer type	Highest level of education completed	Employment status	Completed treatment
1	Male	65-74	Prostate	Master’s degree	Retired	Yes
2	Female	55-64	Breast	Secondary school	Unable to work	No
3	Male	65-74	Esophageal, kidney, and liver	Secondary school	Employed	Yes
4	Male	35-44	Esophageal	Diploma	Unable to work	Yes
5	Female	45-54	Esophageal	Trade, technical, or vocational training	Employed	Yes
6	Female	45-54	Esophageal	Master’s degree	Unable to work	Yes
7	Male	65-74	Esophageal	Bachelor’s degree	Other	Yes
8	Male	65-74	Esophageal and CLL^a^	Master’s degree	Retired	Yes
9	Male	65-74	Hodgkin lymphoma	Bachelor’s degree	Retired	Yes
10	Female	55-64	Breast	Diploma	Employed	No
11	Female	55-64	Breast	Secondary school	Unable to work	No
12	Female	55-64	Breast	Bachelor’s degree	Self-employed	No
13	Female	55-64	Breast	Doctorate degree	Retired	No
14	Male	55-64	Prostate	Diploma	Retired	Yes
15	Male	55-64	RCC^b^ and lung metastases	Diploma	Employed	No
16	Female	35-44	Breast	Master’s degree	Employed	No
17	Female	55-64	Esophageal	Bachelor’s degree	Employed	Yes
18	Female	55-64	Breast	Trade, technical or vocational training	Employed	No

^a^CLL: chronic lymphocytic leukemia.

^b^RCC: renal cell carcinoma.

The mean age of the participants was 58.9 (SD 8.24) years, 56% (10/18) of the participants were female, and 44% (8/18) of the participants were male. A total of 11 (61%) of the 18 participants had completed cancer treatment. All participants reported owning a smartphone and at least 1 other digital device (eg, tablet, laptop, or desktop). In total, 10 (56%) of the 18 participants used activity monitor watches (eg, Fitbit, Garmin, and Apple watch). All participants reported daily use of digital devices, and 33% (6/18) of the respondents expressed a high level of comfort with technology gained through work or leisure activities. The findings of the reflexive thematic analysis were grouped into 5 key themes and 13 subthemes (Textbox 2).

Preferences for specific aspects of telehealth cancer rehabilitation, as reported by participants at any point in the interviews, are presented along with the survey results in Table 4.

**Reflexive thematic analysis themes and subthemes.**

**Themes and subthemes**

1. Telehealth improves accessibility to cancer rehabilitation but is a barrier for others
  Telehealth removes geographical barriers to cancer rehabilitation
  Internet connectivity issues in rural areas
  IT skills
2. Lived experiences of the benefits of telehealth in cancer survivorship
  A more comfortable mode of health care delivery
  Safe and secure care during the pandemic
3. The value of in-person health care delivery
  The desire for personal connection
  Limitations of telehealth
4. Telehealth in cancer care and COVID-19—from novelty to normality
  An enforced and dramatic change
  Now an accepted mode of health care delivery for survivors of cancer
5. The future of telehealth in cancer rehabilitation
  Willingness exists
  Acknowledged need for rehabilitative support
  Amenability of cancer rehabilitation services to telehealth delivery
  Preferences and recommendations for future services

**Table 4:** Preferences for cancer rehabilitation via telehealth (phase 1, survey, and phase 2, semistructured interviews)^a^.

Preference					Survey, n (%)			Semistructured interview, participants reporting
**Delivery of telehealth**								
	Individual consultation			34 (71)			P^b^5, P8, and P12	
	**Group sessions**			N/A^c^			P5, P6, P7, P11, P12, P13, P17, and P18	
		Small group sessions	30 (63)			Nil		
		Larger sessions	25 (52)			Nil		
**Type of telehealth cancer rehabilitation services**								
	Exercise class			30 (63)			P1, P2, P5, P6, P7, P9, P11, P12, P13, and P15	
	**Educational session on**							
		Nutrition	29 (60)			P11		
		Medication management	20 (42)			Nil		
		Fatigue	29 (60)			Nil		
		Sexual well-being	17 (35)			Nil		
		Coping with cancer	23 (48)			P5, P6, P7, P11, P12, P13, P17, and P18		
		Mental health	27 (56)			P1, P2, P4, P6, P8, P11, and P15		
		Lymphoedema	7 (15)			Nil		
		Other	5 (10)			Nil		
**Timing for telehealth cancer rehabilitation**								
	Before treatment			18 (38)			P11 and P12	
	During treatment			23 (48)			Nil	
	Early stage of recovery			36 (75)			P10	
	Survivorship			30 (63)			P1, P5, P6, P11, and P12	
	Palliative care			12 (25)			Nil	

^a^Phase 2 preferences were included if mentioned by respondent at any point in the semistructured interview.

^b^P: participant.

^c^N/A: not applicable.

#### Theme 1: Telehealth Improves Access to Cancer Rehabilitation for Some But Is a Barrier for Others

Participants described that telehealth could improve the equality of access to cancer rehabilitation through its ability to eliminate geographic limitations:

I think accessibility, you don’t have to live in the capital city, to access the right professional, you know, that you can access from anywhere in the country.

Participant 13

People would have travelled across the country to be in the group...but now, the fact that we are online, we have it [all across the country].

Participant 11

However, they also felt that there was a risk of escalating health care inequalities in those who had poor internet connection or poor IT skills. Adequate internet connectivity was deemed an essential facilitator for the delivery of cancer rehabilitation via telehealth. Although connectivity was not an issue for most participants, 2 of them noted poor connections in rural Ireland:

If you’re living in a rural area you’re screwed, because broadband isn’t really up to speed.

Participant 4

Although all participants who completed the semistructured interviews were comfortable with technology, they were concerned that other people, particularly older generations, may not have sufficient confidence, interest, or IT skills to engage with telehealth, highlighting that it may not be suitable for all:

I imagine there is people there who don’t have a clue as to connecting with any of these things.

Participant 2

a lot of people are terrified of technology of an older age...my aunt...she certainly wouldn’t be able to set up an iPad.

Participant 10

#### Theme 2: Lived Experiences of the Benefits of Telehealth in Cancer Survivorship

Most participants reported experiencing ongoing negative sequelae arising from their cancer and its treatments. Participants with ongoing fatigue or pain valued the improved efficiency of health care generated by telehealth because it reduced the time and travel demands of hospital visits:

When I was going through treatment, what I found probably the most exhausting was probably the commuting, so, in and out to appointments and being in queues for appointments.

Participant 10

Engaging in health care appointments from home via telehealth was more comfortable and less physically and mentally tiring:

A huge benefit was that I didn’t have to leave home. I didn’t have to take my break-through meds to travel.

Participant 11

Sometimes people physically, mentally and emotionally would prefer to stay at home.

Participant 17

Participants especially valued that telehealth enabled care to continue without infection risk during COVID-19 and allowed for invaluable group rehabilitative activities to continue even during the strictest periods of lockdown:

Especially these days where you don’t want to be mixing with people, mingling, picking up bugs whatever so it definitely has a place.

Participant 15

Even last year in the heights and the depths of the lockdown a group of us, one of the people was trained or is training in yoga so she started doing zoom yoga.

Participant 17

#### Theme 3: The Value of In-Person Health Care Delivery

Although participants were clearly enthused by the potential of telehealth in cancer rehabilitation, most still highly valued in-person care. There was a strong desire for in-person contact, which facilitated sharing of personal information:

I’d be definitely more inclined to speak intimate things to the doctor in person, rather than over the phone or over Zoom.

Participant 7

Participants discussed how in-person care was still at the core of comprehensive health care. They valued when health care professionals could see their entire body, how they moved, their body language, and emotions:

With the psychotherapy, that (Zoom) really didn’t work...it was all, “Oh yes, everything is fine.” It wasn’t all fine. I only see this lady from the shoulders up, she is not reading my body language.

Participant 12

I don’t think anything can replicate the face to face, the personal...you can read I believe a lot more when you are present with the person.

Participant 17

There was a sense of loss of a less-tangible, but deeply impactful, aspect of in-person care, “the personal touch”:

You are losing the personal touch, seeing the whites of somebody’s eyes.

Participant 15

Participants identified aspects of health care that are not amenable to telehealth delivery:

I do appreciate that certain things can only be done by physical examination.

Participant 10

The downside of seeing the physiotherapist online was that he couldn’t get his hand on (palpate) the spot.

Participant 12

#### Theme 4: Telehealth and COVID-19 (From Novelty to Normality in Cancer Survivorship)

Participants discussed how they were forced to change their mindset about telehealth because of COVID-19, and that for some, support was required to enable the transition in the model of care:

We have been pushed into a situation where people are being forced to use [telehealth].

Participant 18

I think now everything has changed because of COVID. Everything now is about your safety, isn’t it?

Participant 11

The changes in health care delivery using telehealth were acknowledged. Some participants felt that there is a strong willingness in the general public to continue with telehealth service:

On the [telehealth] side of things I think people would grab it with both hands.

Participant 9

I want to avoid queues, I want to avoid commuting, so I personally for me, I think it has been very, very progressive.

Participant 10

Others identified how, with the passing of the emergency phase of the COVID-19 pandemic, there was a returning focus on in-person care and that telehealth options may not be as available:

Actually, what has been talked about with some cancer people I know, regret at how the world is reverting to face to face, closing off the online options.

Participant 13

#### Theme 5: The Future of Telehealth in Cancer Rehabilitation

The participants were clearly enthusiastic about the continued delivery of cancer rehabilitation via telehealth. Participants discussed that any lessons from the recent escalation in telehealth delivery during the pandemic should be brought forward to enhance rehabilitative options for patients living with and beyond cancer:

I may be too enthusiastic about it but I don’t really see any downsides to it. I really just see it as an enormous positive.

Participant 13

There’s an old saying in business, never waste a crisis so whatever you guys have learned about what has worked in the pandemic hold onto it for dear life and don’t roll back on it.

Participant 9

There was acknowledgment that telehealth delivery of cancer rehabilitation is a developing practice, and there is considerable need for further evaluation and implementation of these types of services for survivors of cancer:

I was shocked to see that in the current Slainte Care programme (Irish health care policy document) that has been released that there is not a big focus around cancer and telehealth for cancer patients or cancer society.

Participant 14

Participants highlighted that across the cancer survivorship trajectory, even long into survivorship, individuals may struggle to cope with the physical and psychosocial impairments that occur because of their cancer and its treatments and expressed frustration regarding the lack of rehabilitative support available:

With the COVID thing where you’re not to go out, not to go to crowds, all this thing that you’re at home a lot, just trying to cope with all that at the minute is quite hard.

Participant 2

What I found was very lacking, the mental health end of things.

Participant 11

I think the level of care I was given was excellent, but, what I would say was that aftercare, physically, emotionally, was really lacking.

Participant 16

Participants discussed that many forms of cancer rehabilitation of physical and psychosocial nature could be easily implemented via telehealth:

The other element of the physio would be the exercises to do, post-surgery...I don’t see why they couldn’t be delivered online.

Participant 10

I already do meditation...it’s really, really, good on telehealth (I don’t really want to be in a room with a group of other people when I am closing my eyes).

Participant 2

Despite the overwhelming positive attitude of participants toward telehealth, they highlighted that it is not suitable for all and that some will need support to access telehealth-based health care:

People might need to be eased into it rather than driven into it.

Participant 9

It was important to participants that telehealth technologies be user-friendly and connected across health services:

I think some of it is out there and the problem is it has become a bit fragmented.

Participant 13

Technology, now, mind you, sometimes I would like to throw it in the bin, I know what I need to know and the extra stuff I don’t want to know.

Participant 12

There was a strong desire for reputable and trustworthy information. Participant 10 reported that despite having multiple spinal metastases, “proper medical supervised good information” from a web-based source allowed her to feel protected while doing exercise via telehealth. Some suggested that a hybrid model of rehabilitation would be of benefit to survivors of cancer:

I think we need to move to a hybrid model...People I talk to online, former cancer patients, don’t really want to go back to only face to face.

Participant 13

## Discussion

### Principal Findings

This mixed methods study shows evidence that telehealth-based cancer rehabilitation is broadly acceptable and welcomed by people living with and beyond cancer in Ireland. Participants in both phases of this study deemed telehealth to be highly acceptable for both physical and psychosocial cancer rehabilitation and acknowledged its convenience for this population. Nonetheless, there were some concerns about the limitations of telehealth, particularly regarding accessibility issues, and there was a strong preference among participants to maintain some aspect of in-person care.

A key finding of this study is that there is an important potential role for telehealth in the delivery of cancer rehabilitation. Participants in both phases identified that a wide range of cancer rehabilitation services are amenable to delivery via telehealth, for example, exercise classes, dietetic support, and counseling. Moreover, most phase 1 participants (41/48, 85%) reported experiencing side effects from their cancer and its treatments, which would benefit from rehabilitative input. Up to 40% of survivors of cancer live with long-term posttreatment sequelae including pain, fatigue, and psychosocial issues, and many more individuals experience debilitating short-term side effects, all of which can negatively affect physical function, social engagement, and ultimately quality of life and well-being [32,33]. The impact of these side effects is disproportionally placed on those of lower financial means, experiencing isolation and comorbidities [34]. There is evidently a high requirement for cancer rehabilitation services, especially those that are low cost and easily accessible to people living in isolated circumstances, for example, living in rural areas or without access to transport. However, cancer rehabilitation programs are not the standard of care in many jurisdictions, and existing cancer rehabilitation services are often underresourced and in need of significant investment and expansion to meet demands [35]. Accordingly, new models of care are required to meet these significant demands. Telehealth has the capability to help address these service demands and is widely cited in the literature as a patient-centered means of rehabilitative support that may positively affect functional outcomes [36-38].

The results of the national survey revealed that time and cost savings were popular benefits of telehealth, and during the semistructured interview, respondents elaborated further on this to reveal that the reduction in travel burden was specifically a main benefit of telehealth. Cancer rehabilitation services in Ireland are typically located in major teaching hospitals in urban centers, and the ability to access health care from one’s own home using telehealth was particularly welcomed by phase 2 participants who reported that they were living in rural communities. Globally, there are considerable inequities reported between urban and rural dwelling survivors of cancer [39]. Although the incidence of cancer is typically higher in urban areas, those from more rural communities have an elevated risk of poor health outcomes, with higher levels of cancer-related morbidity and mortality consistently reported [40,41]. These findings may be attributed to the limited availability of clinical care and supportive care services in rural areas, the acknowledged transportation barriers, and the significantly higher financial burden (eg, increased transport costs and increased time away from employment) experienced by rural survivors of cancer. Telehealth provides a unique opportunity to help reduce this health care inequality. Efforts to implement rehabilitative services via telehealth for rural-dwelling survivors of cancer have been warmly received to date. Doorenbos et al [42] previously highlighted how an online support group for rural American Indian and Alaska Native communities survivors of cancer was a viable method of supporting these rurally isolated groups and helped generate a feeling of no longer being alone on the cancer journey. Previous work by our research group [13] also flagged the benefits of telehealth delivery for those from rural communities; in a feasibility study of 12 survivors of esophagogastric cancer who undertook a 12-week multidisciplinary telehealth rehabilitation program, the ability to join sessions from any location with an internet connection was considered very positive and facilitated the participation of patients who lived far away from the urban hospital with minimal disruption to their daily lives. Similarly, Waterland et al [43] recently reported that telehealth was a well-received method of rehabilitation delivery to those in rural and regional areas of Australia about to embark on major cancer surgery. These findings complement the views of our rural participants who welcomed the opportunity to avail cancer rehabilitation without the need to travel to an urban center and highlight the importance of continuing to maintain and develop telehealth cancer rehabilitation services in the postemergency phase of the pandemic.

There was a consensus among participants in both phases of this study that maintaining some level of in-person contact is very important for cancer survivorship care. Concerns remain that telehealth delivery may lead to diminishment of the much-valued patient–health care provider relationship, and a strong recommendation from the semistructured interviews conducted as part of this study was that before commencing cancer rehabilitation via telehealth, patients should have an opportunity to meet in-person with the health care professional to establish their relationship. Similar concerns have been reported in previous work in this field. Recently, Dennett et al [44] reported on the rapid implementation of an exercise-based telehealth rehabilitation program for survivors of cancer. Despite high satisfaction with the rapid care delivery achieved through telehealth, participants felt a sense of loss of meaningful personal connections through this mode of delivery. Indeed, evaluations of telehealth cancer rehabilitation programs consistently report a desire for an element of in-person care to accompany telehealth delivery [45,46]. To this end, the option of a hybrid approach to delivery (ie, a mix of in-person and telehealth delivery) may be an attractive compromise for those survivors of cancer who seek both the benefits of telehealth delivery and in-person care. There is limited literature available on the efficacy of a hybrid approach to cancer rehabilitation, indicating that this topic has been relatively understudied to date. Considerable research is required on how best to deliver cancer rehabilitation in a hybrid mode. Building on our findings from the ReStOre@Home study [13], we will investigate a hybrid approach to cancer rehabilitation in the ReStOre II trial, a randomized controlled trial investigating the efficacy of a 12-week multidisciplinary program of rehabilitation for survivors of upper gastrointestinal and hepatopancreaticobiliary cancers [47].

The results of the survey strongly indicated that telehealth is a welcomed method of delivering a wide variety of cancer rehabilitation services (eg, exercise rehabilitation, fatigue management, and psychological support) in a variety of formats (eg, one-to-one and group-based rehabilitation) across the cancer trajectory from diagnosis to palliative care. When explored more deeply in the semistructured interviews, there was a strong desire for future telehealth cancer rehabilitation services to be delivered in a more effective, inclusive, and patient-centered manner. Moreover, given the unprecedented acceleration of the use of telehealth throughout the COVID-19 pandemic, it is incumbent that any advances in care achieved are maintained, optimized, and used to further improve the equality of access to cancer rehabilitation. The main barriers to the widespread implementation of cancer rehabilitation via telehealth are often because of disparities in internet connectivity, access to devices, and IT knowledge and skills. There is a clear need to minimize these disparities to improve accessibility and maximize inclusion in telehealth-based rehabilitation [48]. Access to high-speed internet is a persistent and prevalent issue, particularly for those living in more rural areas. For example, 2021 figures from the Central Statistics Office in Ireland reported a lower rate of household internet access in the more rural western and northern border areas of Ireland (78% and 75%, respectively), compared with a rate of 93% of households nationally [49]. Lack of access to suitable digital devices can also be a barrier to engaging in telehealth; however, the provision of an IT device by health care or research professionals has been demonstrated as an effective and comparatively affordable method to support inclusion in telehealth interventions [13,50]. Finally, given that only 63% of the world’s population uses the internet, poor digital health literacy is the largest challenge to telehealth engagement [51]. Various educational approaches have been used in the literature to improve digital health knowledge and self-efficacy. These include didactic training, workshops, collaborative learning, and peer tutor models to impart knowledge and improve self-efficacy [52]. Further investigation of these strategies is required to help improve accessibility to telehealth as a means of delivering cancer rehabilitation to a wider cohort of the world’s survivors of cancer.

Drawing on the findings from this study and the related literature described in the discussion above, we compiled a list of recommendations for the design and development of telehealth cancer rehabilitation services. These recommendations are presented in Textbox 3.

**Recommendations for the design and development of telehealth cancer rehabilitation services.**

**Recommendations for the design and development of telehealth cancer rehabilitation services**

1. Providers of cancer rehabilitation services should be supported to develop a telehealth arm to their services, if they are not already doing so.
2. A wide range of cancer rehabilitation specialties and disciplines should consider delivering services via telehealth.
3. Appraise patients’ suitability for telehealth carefully before commencing the intervention. Assess their level of digital skills, internet connectivity, and access to a suitable device.
4. Offer an in-person session for the patient’s first appointment to optimally establish an interpersonal relationship. Encourage and facilitate take-up of this option.
5. Examine how elements of in-person care can be most effectively offered throughout the treatment pathway, for example, develop a hybrid model and provide occasional in-person sessions.
6. Provide an equivalent in-person service for those unable to, or who decline to, use telehealth services.

### Study Strengths and Limitations

The primary strength of this study is its focus on the patients’ voices, which was largely unheard during the rapid change to telehealth services in 2020. Another key strength lies in the robust mixed methods approach. It leveraged the survey findings to shape the interview guide, enabling researchers to delve into important issues with greater depth when engaging with survivors of cancer.

The proportionally low number of in-person surveys completed is because of the restrictions on in-person services during the COVID-19 pandemic. Fewer patients were available in the hospital to be approached for completing the survey, and there were restrictions on interactions with those who were present in person. This limitation has resulted in a high number of responses being gathered on the web, which may be biased toward those who are more comfortable with digital technologies and, therefore, more interested in telehealth. We also acknowledge that all participants in phase 2 were familiar with IT, reporting daily IT use; therefore, there is a need for future research to focus on the viewpoints of those who are less frequent users of IT or those who have difficulty accessing IT and therefore may have differing viewpoints on telehealth. We also note that 10% (5/48) of those surveyed were aged >65 years and that people with breast cancer were disproportionately overrepresented. Future work should focus more on *offline* data collection and specifically seek the opinions of older adults and those with a wider range of cancer diagnoses. We also acknowledge that future studies regarding the development of telehealth services should be inclusive of all stakeholders, especially health care professionals; however, this was beyond the scope of this study, which focused on the patients’ voices.

### Conclusions

Telehealth was widely adopted during the COVID-19 pandemic, and there is now an important opportunity for cancer rehabilitation to develop patient-focused telehealth services. Telehealth is widely accepted and welcomed in cancer rehabilitation, as patients are much more familiar with it now, finding it generally convenient and capable of improving accessibility to rehabilitation services. There is also a strong desire to maintain in-person care for specific circumstances, such as initial assessments or more personal survivorship issues. Those with poor digital skills and poor internet connection must be supported to access telehealth or equivalent in-person care. People living with and beyond cancer will benefit from cancer rehabilitation services that can most appropriately draw from both the “personal touch” of in-person care and the convenience and efficiency of telehealth.

## Abbreviations

TSJCI: Trinity St James’s Cancer Institute
